# Long-term experience with apremilast in patients with psoriatic arthritis: 5-year results from a PALACE 1–3 pooled analysis

**DOI:** 10.1186/s13075-019-1901-3

**Published:** 2019-05-10

**Authors:** Arthur Kavanaugh, Dafna D. Gladman, Christopher J. Edwards, Georg Schett, Benoit Guerette, Nikolay Delev, Lichen Teng, Maria Paris, Philip J. Mease

**Affiliations:** 1 School of Medicine, University of California San Diego, 9500 Gilman Drive, La Jolla, CA 92037 USA; 2 Division of Rheumatology, Krembil Research Institute, Toronto Western Hospital, University of Toronto, Toronto, Ontario Canada; 3 NIHR Wellcome Trust Clinical Research Facility, University Hospital Southampton, Southampton, UK; 4 Friedrich-Alexander-Universität Erlangen-Nürnberg und Universitätsklinikum Erlangen, Erlangen, Germany; 5 Celgene Corporation, Summit, NJ USA; 6 Rheumatology Clinical Research Division, Swedish Medical Center/Providence St. Joseph Health and University of Washington, Seattle, WA USA

**Keywords:** Apremilast, Drug safety, Psoriatic arthritis, Treatment efficacy

## Abstract

**Background:**

The efficacy and safety of apremilast were assessed in patients with psoriatic arthritis (PsA) in three phase III clinical trials with similar designs (PALACE 1, 2, and 3).

**Methods:**

Following a 24-week, randomized (1:1:1 to apremilast 30 mg twice daily, 20 mg twice daily, or placebo), double-blind phase and a 28-week blinded active treatment phase, patients could receive apremilast in open-label extension studies for an additional 4 years. Eligible adult patients had active PsA for ≥ 6 months and three or more swollen joints and three or more tender joints despite prior treatment with disease-modifying anti-rheumatic drugs.

**Results:**

A total of 1493 randomized patients received one or more doses of study medication (placebo: *n* = 496; apremilast 30 mg twice daily: *n* = 497; apremilast 20 mg twice daily: *n* = 500). In patients continuing apremilast treatment, response was sustained without new safety issues. At week 260, 67.2% of remaining patients achieved an ACR20 response, and 44.4% and 27.4% achieved ACR50 and ACR70 responses, respectively. Among patients with baseline enthesitis and dactylitis, 62.4% achieved a Maastricht Ankylosing Spondylitis Enthesitis Score of 0 and 80.9% achieved a dactylitis count of 0, respectively. In patients who had ≥ 3% baseline psoriasis body surface area involvement, 43.6% achieved ≥ 75% reduction from the baseline Psoriasis Area and Severity Index scores. The most commonly reported adverse events (AEs) were diarrhea, nausea, headache, upper respiratory tract infection, and nasopharyngitis, with most diarrhea and nausea AEs occurring within the first 2 weeks of treatment and usually resolving within 4 weeks. Reported rates of depression during the study were low (≤ 1.8%). The majority of patients maintained their weight within 5% of baseline during the study. No new safety concerns or increases in the incidence or severity of AEs were observed over the long term.

**Conclusions:**

Apremilast maintained clinical benefit and a favorable safety profile for up to 5 years among patients with PsA.

**Trial registration:**

ClinicalTrials.gov NCT01172938, NCT01212757, NCT01212770

**Electronic supplementary material:**

The online version of this article (10.1186/s13075-019-1901-3) contains supplementary material, which is available to authorized users.

## Introduction

Apremilast, an oral phosphodiesterase 4 inhibitor, has been proven effective in patients with psoriatic arthritis (PsA) [1–3]. Apremilast was studied for the treatment of PsA in three similarly designed, phase III studies (Psoriatic Arthritis Long-term Assessment of Clinical Efficacy [PALACE] 1, 2, and 3) that enrolled disease-modifying anti-rheumatic drug (DMARD)-experienced patients [1–3]. These studies included an open-label extension phase to evaluate the effects of long-term exposure to apremilast. We now report the final long-term findings for PALACE 1, 2, and 3 in patients with active PsA who received apremilast for up to 260 weeks.

## Methods

### Study design

PALACE 1, 2, and 3 were phase III, multicenter, randomized, double-blind, placebo-controlled studies. The three studies used the same study design and have been previously described [1–3]. Briefly, each study comprised a 24-week, randomized, double-blind, placebo-controlled phase; a 28-week blinded active treatment phase; and a long-term, open-label extension phase for up to an additional 4 years. Patients were randomly assigned at baseline (1:1:1) to receive placebo, apremilast 30 mg twice daily, or apremilast 20 mg twice daily. Patients randomized to placebo were re-randomized to apremilast 30 mg twice daily or 20 mg twice daily at week 16 (early escape) or week 24. Patients who completed 52 weeks of treatment were eligible to enroll in the long-term extension for apremilast treatment for an additional 4 years.

### Patients

As previously described, patients were adults with active PsA for ≥ 6 months who met the Classification Criteria for Psoriatic Arthritis (CASPAR) [4] and had three or more swollen joints and three or more tender joints despite prior exposure to conventional or biologic DMARDs [1–3]. Patients enrolled in PALACE 3 also had active skin disease with at least one plaque psoriasis lesion that was ≥ 2 cm in size [3].

### Study outcomes

#### Efficacy assessments

Efficacy endpoints included rates of patients achieving ≥ 20% improvement in American College of Rheumatology response criteria (ACR20) (primary endpoint), ACR50, and ACR70 responses, modified for PsA using the 76 swollen joint count (SJC) and 78 tender joint count (TJC) (i.e., inclusion of distal interphalangeal joints of the toes and carpometacarpal joints to total joint counts) [5, 6]; changes from baseline in SJC and TJC; proportions of patients achieving a Maastricht Ankylosing Spondylitis Enthesitis Score (MASES) [7] of 0 among those with enthesitis at baseline, and proportions of patients achieving a dactylitis count of 0 among those with dactylitis at baseline. Physical function assessments included mean change from baseline and achievement of a minimal clinically important difference (MCID) of ≥ 0.35 [8] in the Health Assessment Questionnaire-Disability Index (HAQ-DI) score [9]. Skin involvement was also assessed using ≥ 75% reduction from the baseline Psoriasis Area and Severity Index (PASI-75) in patients with psoriasis involving ≥ 3% of body surface area at baseline.

#### Safety assessments

Safety assessments were conducted at scheduled visits during each treatment phase and in the event of early termination/withdrawal and included collection of adverse events (AEs) as well as physical examination and clinical laboratory findings. The AEs occurring after randomization were classified using the Medical Dictionary for Drug Regulatory Activities Classification System.

### Statistical analyses

Data were pooled across the three studies. Efficacy data were analyzed descriptively by time point using all available data; analyses through week 260 were based on observed data without imputation for missing data. Safety outcomes were analyzed descriptively for all patients who received one or more doses of apremilast and are presented for the apremilast-exposure periods from weeks 0 to ≤ 52 (relative to the first dose of apremilast), weeks > 52 to ≤ 104, weeks > 104 to ≤ 156, weeks > 156 to ≤ 208, and weeks > 208.

## Results

### Patients

Across PALACE 1–3, 1493 patients were randomized and received one or more doses of study medication (placebo: *n* = 496; apremilast 30 mg twice daily: *n* = 497; apremilast 20 mg twice daily: *n* = 500). Baseline patient demographic and disease characteristics have been previously described [1–3] and were comparable across treatment groups. Among patients randomized to apremilast 30 mg or 20 mg at baseline, 44.5% (221/497) and 42.4% (212/500) completed week 260, respectively. Among patients randomized to placebo at baseline and switched to apremilast 30 mg or 20 mg at week 16 or 24, 49.1% (110/224) and 41.8% (92/220) completed week 260, respectively. Of those who received apremilast 30 mg or 20 mg entering week 52, regardless of when initiated (baseline, week 16, or week 24), 63.2% (331/524) and 58.7% (304/518) completed 260 weeks, respectively. A total of 684 patients had > 208 weeks of exposure to apremilast (20 mg, *n* = 320; 30 mg, *n* = 364). Over the apremilast-exposure period, the most commonly cited reasons for study discontinuation were withdrawal of consent by patient (18.0%), lack of efficacy (17.6%), and adverse event (11.9%). Other reasons for discontinuation included lost to follow-up (2.6%), non-compliance (1.2%), protocol violation (0.6%), death (0.5%), and “other” (3.3%).

### Efficacy outcomes

**Fig. 1:**
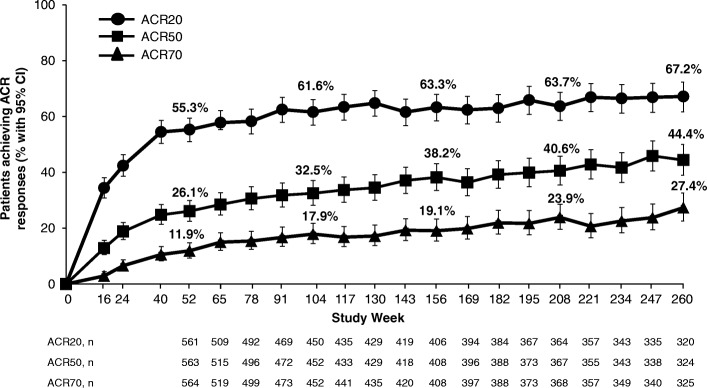
ACR responses in PsA patients receiving apremilast 30 mg up to 260 weeks. Data as observed. The analysis includes all patient data, including the placebo-controlled phase, regardless of when the patients started taking apremilast (baseline, week 16, or week 24). The proportions of psoriatic arthritis (PsA) patients achieving ACR20, ACR50, or ACR70 responses at study visits up to week 260 are shown. Error bars represent 95% confidence interval (CI). *n* represents the number of patients with data available at that time point

**Fig. 2:**
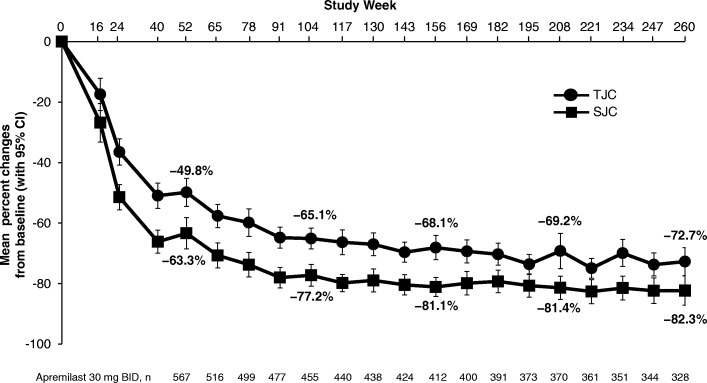
SJC/TJC improvements in PsA patients receiving apremilast 30 mg up to 260 weeks. Data as observed. The analysis includes all patient data, including the placebo-controlled phase, regardless of when the patients started taking apremilast (baseline, week 16, or week 24). The mean percentage changes in swollen joint count (SJC) and tender joint count (TJC) for psoriatic arthritis (PsA) patients at study visits up to week 260 are shown. Error bars represent 95% confidence interval (CI). *n* represents the number of patients with data available at that time point

Of patients receiving apremilast 30 mg twice daily, 55.3% achieved an ACR20 response at week 52; at week 260, 67.2% of patients who continued apremilast treatment achieved an ACR20 response. Similarly, ACR50 and ACR70 responses were sustained over 260 weeks with continued treatment (Fig. 1, Additional file 1: Table S1). Mean SJC and TJC improved by 63.3% and 49.8% at week 52, with improvements reaching 82.3% and 72.7%, respectively, with continued treatment at week 260 (Fig. 2, Additional file 1: Table S1).

**Fig. 3:**
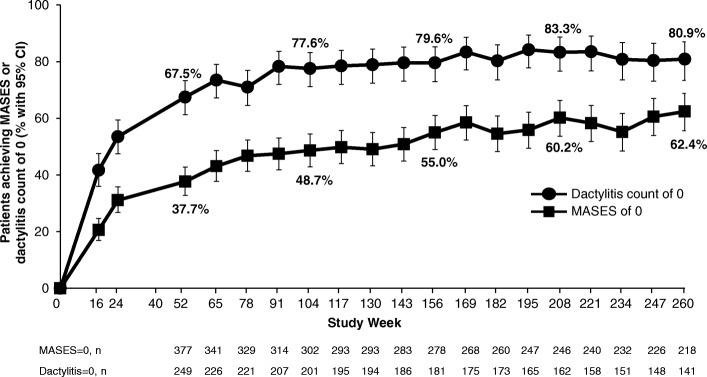
Enthesitis/dactylitis improvements in PsA patients receiving apremilast 30 mg up to 260 weeks. Data as observed. The analysis includes all patient data, including the placebo-controlled phase, regardless of when the patients started taking apremilast (baseline, week 16, or week 24). The proportions of patients achieving a Maastricht Ankylosing Spondylitis Enthesitis Score (MASES) of 0 (> 0 indicating enthesitis) or a dactylitis count of 0 at study visits up to week 260 are shown. Error bars represent 95% confidence interval (CI). *n* represents the number of patients with either MASES ≥ 1 or dactylitis count ≥ 1 at baseline and data available at that time point

Among patients with enthesitis or dactylitis at baseline, mean changes in MASES and dactylitis at week 260 were − 2.9 and − 2.8, respectively. The proportions of those achieving a MASES of 0 or a dactylitis count of 0 increased over 52 weeks and were maintained through week 260 with continued apremilast 30 mg treatment (Fig. 3, Additional file 1: Table S1).

**Fig. 4:**
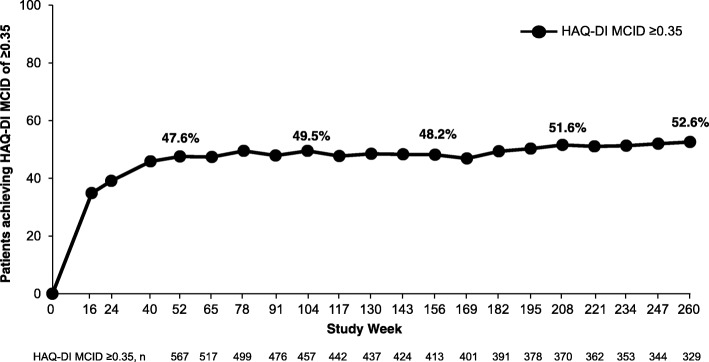
Improvements in physical function among PsA patients receiving apremilast 30 mg up to 260 weeks. Data as observed. The analysis includes all patient data, including the placebo-controlled phase, regardless of when the patients started taking apremilast (baseline, week 16, or week 24). The proportions of patients achieving a Health Assessment Questionnaire-Disability Index (HAQ-DI) minimal clinically important difference (MCID) of ≥ 0.35 at study visits up to week 260 are shown. *n* represents the number of patients with data available at that time point

Improvements in physical function were maintained through week 260 in patients who continued receiving apremilast 30 mg twice daily, including mean change in HAQ-DI and the proportion achieving a HAQ-DI MCID ≥ 0.35 (Fig. 4, Additional file 1: Table S1).

Among patients with plaque psoriasis involving ≥ 3% of the body surface area at baseline, the proportion of patients achieving PASI-75 response was generally maintained with continued treatment, with 43.6% of patients having a PASI-75 response at week 260 (Additional file 1: Figure S1 and Additional file 1: Table S1).

Pooled results for apremilast 30 mg twice daily were consistent with the results observed in the individual studies for all outcomes, including ACR20 responses (Additional file 1: Figure S2). Apremilast 20 mg twice daily also demonstrated improvements in the signs and symptoms of PsA (Additional file 1: Table S1).

### Safety outcomes

Most AEs were mild or moderate in severity with both apremilast doses over weeks 0 to ≤ 52. During weeks 0 to ≤ 52, AEs occurring in ≥ 5% of apremilast-exposed patients included diarrhea, nausea, headache, upper respiratory tract infection, and nasopharyngitis. Most diarrhea and nausea AEs were reported within the first 2 weeks of treatment and usually resolved within 4 weeks; the frequency of gastrointestinal AEs decreased with longer apremilast exposure, and the frequency of other common AEs decreased or remained stable with prolonged exposure (Table 1).

**Table 1:** Summary of safety in patients with PsA through 260 weeks of apremilast treatment by treatment period

Patients, *n* (%)	Apremilast-exposure period*									
	Weeks 0 to ≤ 52		Weeks > 52 to ≤ 104		Weeks > 104 to ≤ 156		Weeks > 156 to ≤ 208		Weeks > 208 to ≤ 260	
	30 mg twice daily (*n* = 721)	20 mg twice daily (*n* = 720)	30 mg twice daily (*n* = 520)	20 mg twice daily (*n* = 508)	30 mg twice daily (*n* = 443)	20 mg twice daily (*n* = 422)	30 mg twice daily (*n* = 401)	20 mg twice daily (*n* = 366)	30 mg twice daily (*n* = 364)	20 mg twice daily (*n* = 320)
Patients with										
≥ 1 AE	526 (73.0)	507 (70.4)	316 (60.8)	326 (64.2)	287 (64.8)	273 (64.7)	238 (59.4)	219 (59.8)	179 (49.2)	188 (58.8)
≥ 1 serious AE	47 (6.5)	40 (5.6)	35 (6.7)	39 (7.7)	40 (9.0)	33 (7.8)	27 (6.7)	28 (7.7)	21 (5.8)	21 (6.6)
≥ 1 serious infection	6 (0.8)	3 (0.4)	5 (1.0)	8 (1.6)	4 (0.9)	5 (1.2)	4 (1.0)	5 (1.4)	7 (1.9)	5 (1.6)
AE leading to study withdrawal	57 (7.9)	52 (7.2)	13 (2.5)	11 (2.2)	7 (1.6)	10 (2.4)	8 (2.0)	11 (3.0)	5 (1.4)	2 (0.6)
Death	0 (0.0)	1^§^ (0.1)	1^‡^ (0.2)	0 (0.0)	0 (0.0)	0 (0.0)	2^||#^ (0.5)	1^¶^ (0.3)	1** (0.3)	0 (0.0)
AEs occurring in ≥ 5% of any treatment group/period										
Diarrhea	113 (15.7)	89 (12.4)	20 (3.8)	10 (2.0)	12 (2.7)	13 (3.1)	5 (1.2)	3 (0.8)	2 (0.5)	6 (1.9)
Nausea	108 (15.0)	69 (9.6)	11 (2.1)	8 (1.6)	10 (2.3)	4 (0.9)	3 (0.7)	6 (1.6)	1 (0.3)	4 (1.3)
Headache	75 (10.4)	61 (8.5)	17 (3.3)	15 (3.0)	12 (2.7)	11 (2.6)	8 (2.0)	9 (2.5)	2 (0.5)	8 (2.5)
URTI	60 (8.3)	71 (9.9)	27 (5.2)	40 (7.9)	24 (5.4)	30 (7.1)	22 (5.5)	27 (7.4)	21 (5.8)	18 (5.6)
Nasopharyngitis	41 (5.7)	48 (6.7)	31 (6.0)	29 (5.7)	20 (4.5)	30 (7.1)	27 (6.7)	26 (7.1)	24 (6.6)	22 (6.9)
Select laboratory assessments, n/m (%)										
ALT, > 3× ULN	9/713 (1.3)	8/713 (1.1)	2/518 (0.4)	1/502 (0.2)	2/442 (0.5)	2/419 (0.5)	1/401 (0.2)	2/364 (0.5)	1/363 (0.3)	4/319 (1.3)
Creatinine, > 1.7× ULN, μmol/L	1/713 (0.1)	1/713 (0.1)	0/518 (0.0)	0/502 (0.0)	0/442 (0.0)	1/419 (0.2)	1/401 (0.2)	4/364 (1.1)	1/363 (0.3)	3/319 (0.9)
Hemoglobin < 10.5 g/dL (male) or < 8.5 g/dL (female)	5/713 (0.7)	5/712 (0.7)	4/517 (0.8)	0/503 (0.0)	5/442 (1.1)	2/419 (0.5)	5/401 (1.2)	2/364 (0.5)	5/362 (1.4)	1/319 (0.3)
Leukocytes < 1.5 × 10^9^/L	0/713 (0.0)	0/712 (0.0)	0/517 (0.0)	0/503 (0.0)	0/442 (0.0)	0/419 (0.0)	0/401 (0.0)	0/364 (0.0)	0/362 (0.0)	0/319 (0.0)
Neutrophils < 1 × 10^9^ L	2/713 (0.3)	4/712 (0.6)	3/517 (0.6)	2/502 (0.4)	2/442 (0.5)	1/419 (0.2)	2/401 (0.5)	2/364 (0.5)	4/362 (1.1)	1/319 (0.3)
Platelets < 75 × 10^9^/L	0/713 (0.0)	0/712 (0.0)	0/517 (0.0)	1/503 (0.2)	1/441 (0.2)	1/419 (0.2)	0/399 (0.0)	0/364 (0.0)	2/362 (0.6)	0/319 (0.0)

*AE* adverse event, *URTI* upper respiratory tract infection

*Includes all patients who received apremilast during the time interval relative to the start of apremilast treatment

^§^Multiorgan failure not suspected to be treatment-related

^‡^Motor vehicle accident on study day 489

^||^Cerebrovascular accident on day 1330 in a 69-year-old man, considered unrelated to the study drug; patient had a history of myocardial infarction, atrial fibrillation, and cerebrovascular accident

^#^Stroke on day 1224 in a 58-year-old woman, considered unrelated to the study drug; patient had a history of chronic ischemic heart disease, hypertension, alcoholism, and atrial fibrillation

^¶^Heart failure on day 1462 in a 70-year-old man, considered unrelated to the study drug; patient had a history of ischemic heart disease, arrhythmia, and heart failure

**Necrotizing fasciitis of the anterior abdominal wall, refractory hypotensive shock, and acute renal failure considered not related to apremilast by investigator; patient had a history of diabetes mellitus

No new safety concerns or increases in the incidence or severity of AEs were seen with exposure over 260 weeks (Table 1). Discontinuations due to AEs during weeks 0 to ≤ 52 occurred in 7.6% of combined apremilast patients. In the longer exposure periods, ≤ 2.5% of patients discontinued treatment because of AEs. The most frequently reported AEs leading to discontinuation of apremilast were diarrhea, nausea, and headache and were primarily reported during the 0- to ≤ 52-week period (Table 2).

**Table 2:** Adverse events leading to discontinuation in > 2 patients in any single exposure period through 260 weeks of apremilast treatment by treatment period

Patients, *n* (%)	Apremilast-exposure period*									
	Weeks 0 to ≤ 52		Weeks > 52 to ≤ 104		Weeks > 104 to ≤ 156		Weeks > 156 to ≤ 208		Weeks > 208 to ≤ 260	
	30 mg twice daily (*n* = 721)	20 mg twice daily (*n* = 720)	30 mg twice daily (*n* = 520)	20 mg twice daily (*n* = 508)	30 mg twice daily (*n* = 443)	20 mg twice daily (*n* = 422)	30 mg twice daily (*n* = 401)	20 mg twice daily (*n* = 366)	30 mg twice daily (*n* = 364)	20 mg twice daily (*n* = 320)
Nausea	16 (2.2)	8 (1.1)	0 (0.0)	0 (0.0)	0 (0.0)	0 (0.0)	0 (0.0)	0 (0.0)	0 (0.0)	0 (0.0)
Diarrhea	15 (2.1)	6 (0.8)	1 (0.2)	0 (0.0)	0 (0.0)	1 (0.2)	0 (0.0)	0 (0.0)	1 (0.3)	0 (0.0)
Headache	11 (1.5)	4 (0.6)	0 (0.0)	0 (0.0)	1 (0.2)	0 (0.0)	0 (0.0)	0 (0.0)	0 (0.0)	0 (0.0)
Vomiting	6 (0.8)	1 (0.1)	0 (0.0)	0 (0.0)	0 (0.0)	0 (0.0)	0 (0.0)	0 (0.0)	0 (0.0)	0 (0.0)
Dizziness	4 (0.6)	2 (0.3)	0 (0.0)	0 (0.0)	0 (0.0)	0 (0.0)	0 (0.0)	0 (0.0)	0 (0.0)	0 (0.0)
Upper abdominal pain	3 (0.4)	4 (0.6)	0 (0.0)	0 (0.0)	0 (0.0)	0 (0.0)	0 (0.0)	1 (0.3)	0 (0.0)	0 (0.0)
Migraine	3 (0.4)	1 (0.1)	0 (0.0)	0 (0.0)	0 (0.0)	0 (0.0)	0 (0.0)	0 (0.0)	0 (0.0)	0 (0.0)
Fatigue	3 (0.4)	2 (0.3)	0 (0.0)	0 (0.0)	0 (0.0)	0 (0.0)	0 (0.0)	1 (0.3)	0 (0.0)	0 (0.0)

*Includes all patients who received apremilast during the time interval relative to the start of apremilast treatment

Serious AEs occurred at low and similar rates (≤ 8.4% of combined apremilast patients) during each of the five apremilast-exposure periods. Infections and infestations and musculoskeletal and connective tissue disorders were the most common classes of serious AE. Each was reported in ≤ 1.8% of combined apremilast patients during each apremilast-exposure period. During the placebo-controlled period, rates of major cardiac events, malignant neoplasms, and serious opportunistic infections were comparable between placebo and apremilast [1–3]. There was no increase in overall rates with longer-term exposure.

In the placebo-controlled period, reports of depression were rare but greater with apremilast compared with placebo (1.2% vs 0.8%). Rates of depression during the long-term study were low with combined rates of 1.7%, 1.8%, 1.5%, 1.6%, and 1.0% for apremilast reported across the five periods examined. No completed suicides occurred during the study. Attempted suicide was reported by one patient during the weeks 0 to ≤ 52 apremilast-exposure period (following a serious family altercation) and one patient during the weeks > 52 to ≤ 104 apremilast-exposure period (history of depression, bipolar affective disorder, physical/emotional abuse). No suicide attempts were reported during the remaining apremilast-exposure periods. The ranges of mean (median) weight changes were between − 1.34 kg (− 1.00 kg) and − 0.92 kg (− 0.90 kg) with apremilast 30 mg and − 1.36 kg (− 1.00 kg) and − 0.98 kg (− 0.40 kg) with apremilast 20 mg across exposure periods, with the majority of patients maintaining their weight within 5% of baseline during the study. Weight loss > 5% was observed in 21.8% (155/711) of apremilast 30 mg patients and 21.0% (149/708) of apremilast 20 mg patients. Clinically important laboratory abnormalities were generally infrequent, transient, and of similar incidence during all periods (Table 1). Throughout the longer-term apremilast-exposure periods, the majority of patients with normal baseline values for laboratory parameters evaluated continued to maintain values within the normal range (Additional file 1: Table S2).

## Discussion

In one of the largest cohorts of patients with active PsA, treatment with apremilast was evaluated for up to 5 years in the phase III PALACE 1, 2, and 3 studies. More than 40% of patients continued apremilast treatment in the long-term extension studies through 260 weeks.

During the long-term open-label extension phases of these studies, the population of patients who continued apremilast treatment for 5 years maintained improvements in signs and symptoms of PsA, including SJC, TJC, and physical function. Enthesitis, dactylitis, and psoriasis were also improved in patients with these manifestations at baseline. Given that one third of patients remaining on treatment at week 260 did not achieve an ACR20 response, results suggest using outcome measures other than ACR20 response criteria when evaluating PsA disease activity. In a separate study characterizing the clinical benefits associated with long-term apremilast treatment in patients who did not achieve an ACR20 response at week 104, significant improvements in core psoriatic domains were observed despite the ACR20 non-response [10, 11]. Taken together, this may explain why patients who fail to achieve an ACR20 response continue on long-term apremilast treatment.

Apremilast continued to demonstrate a favorable safety profile in patients with active PsA who were previously treated with a DMARD and/or biologic therapies. The rates and types of AEs were consistent throughout the three PALACE studies, and no new safety signals were observed across 5 years of apremilast treatment.

A limitation of these studies is that the impact of apremilast on structural disease progression was not assessed in the PALACE clinical trial program. Importantly, the results from this analysis indicated significant improvements in the numbers of swollen and tender joints over 5 years of treatment. Given that improvements in joint inflammation have been associated with the prevention of structural damage [12], it is possible that high levels of disease control among patients in the PALACE 1–3 studies may have been associated with inhibition of disease progression. A limitation of controlled clinical studies is the enrollment of patients with restricted eligibility criteria, which may not represent the general population of patients with PsA. Additionally, although open-label extensions offer insight into the experience with a given treatment in the population of patients who do remain on therapy over the long term, efficacy results may be biased by the discontinuation of patients due to the lack or loss of response and absence of a control arm. Emerging real-world evidence will provide more insight into the use and effectiveness of apremilast in clinical practice.

## Conclusions

In this pooled analysis of three long-term extension studies, apremilast continued to demonstrate a favorable safety profile, with no new safety concerns identified, and was generally well tolerated for up to 5 years. Patients who continued therapy demonstrated sustained, clinically meaningful improvements in the signs and symptoms across various domains of PsA as well as in physical function.

## Additional file

Additional file 1: **Figure S1.** PASI-75 response in PsA patients receiving apremilast 30 mg twice daily up to 260 weeks. **Figure S2.** ACR20 responses in PsA patients receiving apremilast 30 mg twice daily up to 260 weeks across PALACE studies. **Table S1.** Efficacy outcomes at week 260 in patients with PsA treated with apremilast. **Table S2.** Clinically important shifts in select laboratory measurements among patients with normal values at baseline. (DOCX 117 kb)

## Abbreviations

AE: Adverse event
DMARD: Disease-modifying anti-rheumatic drug
HAQ-DI: Health Assessment Questionnaire-Disability Index
MASES: Maastricht Ankylosing Spondylitis Enthesitis Score
PALACE: Psoriatic Arthritis Long-term Assessment of Clinical Efficacy
PASI-75: ≥ 75% reduction from the baseline Psoriasis Area and Severity Index score
PsA: Psoriatic arthritis
SJC: Swollen joint count
TJC: Tender joint count
