# Supplementary material for: Long-term experience with apremilast in patients with psoriatic arthritis: 5-year results from a PALACE 1–3 pooled analysis
Source: Arthritis Res Ther. 2019 May 10;21:118. doi: 10.1186/s13075-019-1901-3 (PMC6509758; doi:10.1186/s13075-019-1901-3)
Supplement: Supplementary file 1 — Figure S1. PASI-75 response in PsA patients receiving apremilast 30 mg twice daily up to 260 weeks. Figure S2. ACR20 responses in PsA patients receiving apremilast 30 mg twice daily up to 260 weeks across PALACE studies. Table S1. Efficacy outcomes at week 260 in patients with PsA treated with apremilast. Table S2. Clinically important shifts in select laboratory measurements among patients with normal values at baseline. (DOCX 117 kb) [file 13075_2019_1901_MOESM1_ESM.docx]

**Supplementary Material**

**Efficacy and safety of apremilast treatment continued for up to 5 years in patients with psoriatic arthritis: a PALACE 1-3 pooled analysis**

Arthur Kavanaugh, Dafna D. Gladman, Christopher J. Edwards, Georg Schett,
Benoit Guerette, Nikolay Delev, Lichen Teng, Maria Paris, Philip J. Mease

**Fig S1** PASI-75 response in PsA patients receiving apremilast 30 mg twice daily up to 260 weeks

**Fig S2** ACR20 responses in PsA patients receiving apremilast 30 mg twice daily up to 260 weeks across PALACE studies.

**Table S1.** Efficacy outcomes at Week 260 in patients with PsA treated with apremilast

**Table S2.** Clinically important shifts in select laboratory measurements among patients with normal values at baseline

**Fig S1** PASI-75 response in PsA patients receiving apremilast 30 mg twice daily up to 260 weeks


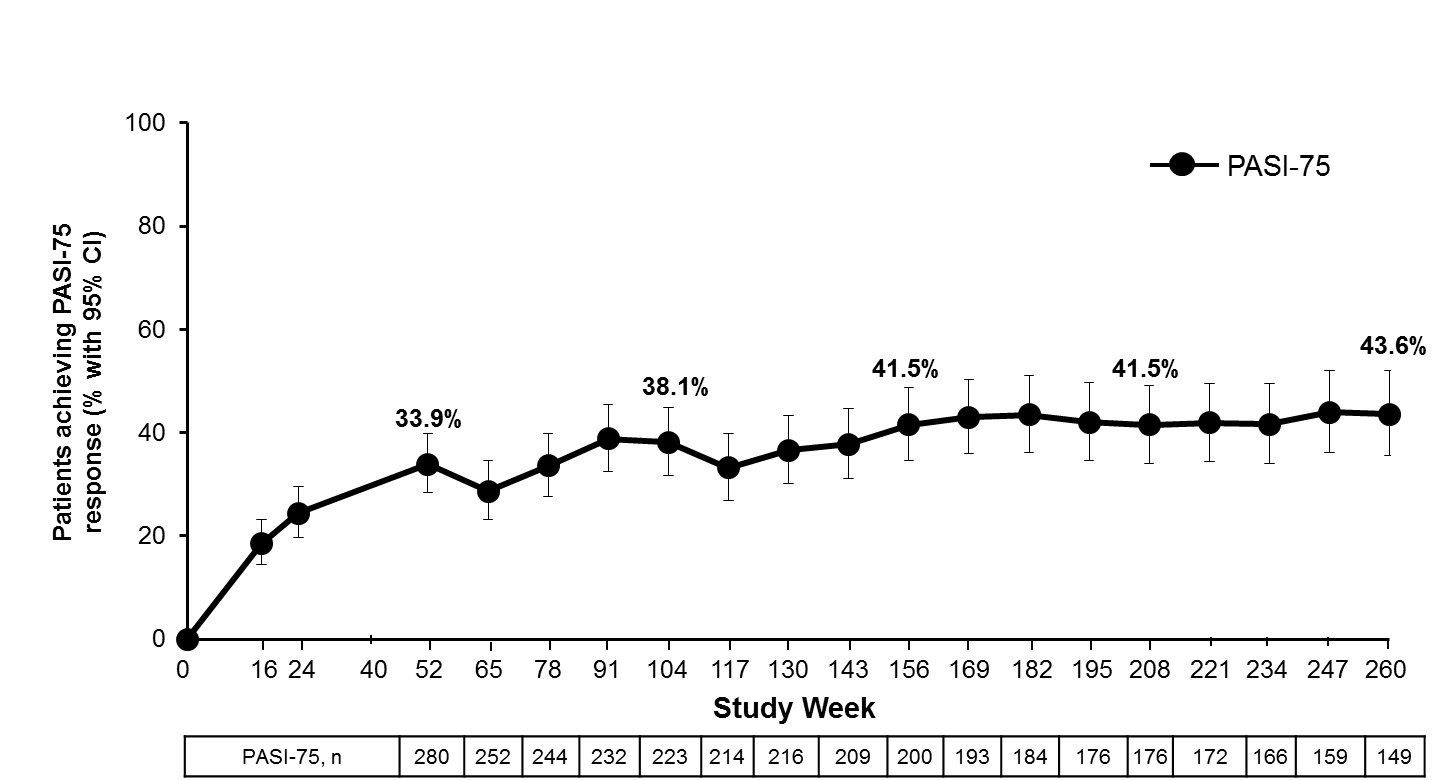


Data as observed. Analysis includes all patient data, including the placebo-controlled phase, regardless of when patients started taking apremilast (baseline, Week 16, or Week 24). The proportions of psoriatic arthritis (PsA) patients achieving ≥75% reduction from baseline Psoriasis Area and Severity score (PASI-75) at study visits up to Week 260 are shown. Error bars represent 95% confidence interval (CI). The n represents the number of patients with psoriasis body surface area involvement ≥3% at baseline and with data available at the time point.

**Fig S2** ACR20 responses in PsA patients receiving apremilast 30 mg twice daily up to 260 weeks across PALACE studies


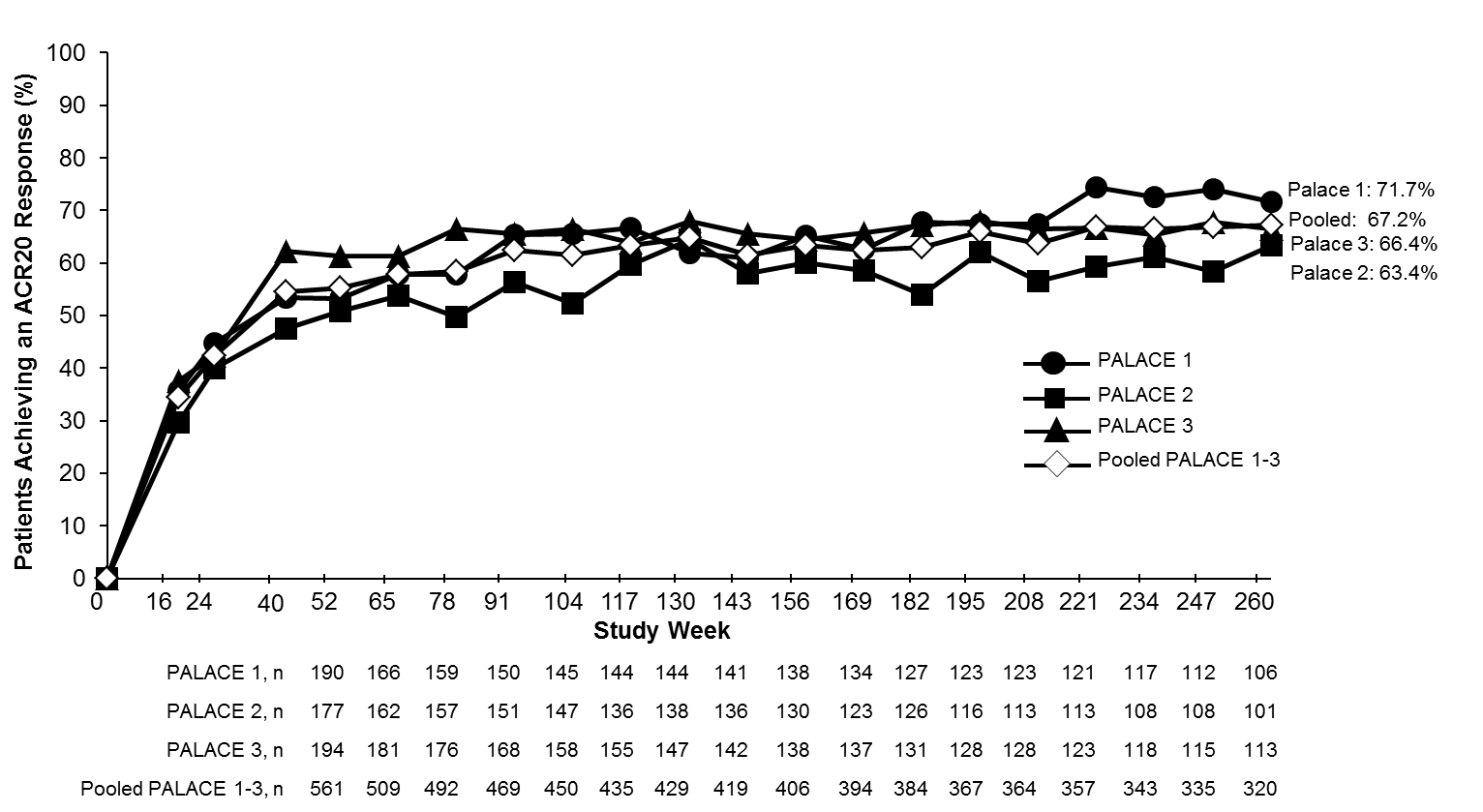


Data as observed. Analyses include all patient data, including the placebo-controlled phase, regardless of when patients started taking apremilast (baseline, Week 16, or Week 24) for PALACE 1 (circles), PALACE 2 (squares) or PALACE 3 (triangles) and pooled across all studies (diamonds). The n represents the number of patients with data available at the time point; it may vary slightly for each outcome. ACR20, ≥20% improvement in American College of Rheumatology Criteria; n, number of patients with data available; PsA, psoriatic arthritis.

**Table S1.** Efficacy outcomes at Week 260 in patients with PsA treated with apremilast

| Efficacy outcome | Apremilast 30 mg twice daily (*n* = 329) | Apremilast 20 mg twice daily (*n* = 304) |
| --- | --- | --- |
| ACR responses, n/N1 (%) [95% CI] ACR20 ACR50 ACR70 | 215/320 (67.2) [61.7, 72.3] 144/324 (44.4) [39.0, 50.0] 89/325 (27.4) [22.6, 32.6] | 202/298 (67.8) [62.2, 73.1] 142/297 (47.8) [42.0, 53.7] 76/300 (25.3) [20.5, 30.7] |
| Joint counts, mean % change from baseline (SD) SJC TJC | −82.3 (44.5) −72.7 (42.8) | −86.0 (29.1) −75.1 (41.2) |
| MASES of 0*****, n/N1 (%) [95% CI] | 136/218 (62.4) [55.6, 68.8] | 117/182 (64.3) [56.9, 71.2] |
| Dactylitis count of 0^§^, n/N1 (%) [95% CI] | 114/141 (80.9) [73.4, 87.0] | 114/131 (87.0) [80.0, 92.3] |
| HAQ-DI Change from baseline, mean (SD) Achieved MCID, n/N1 (%) | −0.42 (0.63) 173/329 (52.6) | −0.37 (0.63) 146/303 (48.2) |
| PASI-75 response**^‡^**, n/N1 (%) [95% CI] | 65/149 (43.6) [35.5, 52.0] | 56/136 (41.2) [32.8, 49.9] |

*****For subjects with enthesitis at baseline. ^§^For subjects with dactylitis at baseline. **^‡^**For subjects with psoriasis involving ≥3% for body surface area at baseline.

*Abbreviations:* *CI* confidence interval; *HAQ-DI* Health Assessment Questionnaire-Disability Index; *MASES* Maastricht Ankylosing Spondylitis Enthesitis Score; *MCID* minimal clinically important difference; *n* number of patients with response; *N1* number of patients with data available; *PsA* psoriatic arthritis; *SD* standard deviation; *SJC* swollen joint count; *TJC* tender joint count.

**Table S2.** Clinically important shifts in select laboratory measurements among patients with normal values at baseline

| Laboratory parameter | Shift | Apremilast 30 mg twice daily and 20 mg twice daily exposure period* | | | | |  |
| --- | --- | --- | --- | --- | --- | --- | --- |
|  |  | ≤1 year | >1 to ≤2 years | >2 to ≤3 years | >3 to ≤4 years | >4 years | |
|  |  | Patients shifted, m/n (%)^†^ | Patients shifted, m/n (%)^†^ | Patients shifted, m/n (%)^†^ | Patients shifted, m/n (%)^†^ | Patients shifted, m/n (%)^†^ | |
| ALT | Normal to  high | 43/1279 (3.4) | 38/915 (4.2) | 25/777 (3.2) | 30/686 (4.4) | 26/611 (4.3) | |
| AST | Normal to  high | 47/1326 (3.5) | 39/955 (4.1) | 29/807 (3.6) | 32/714 (4.5) | 52/635 (8.2) | |
| Creatinine | Normal to  high | 31/1311 (2.4) | 35/938 (3.7) | 42/797 (5.3) | 39/710 (5.5) | 36/638 (5.6) | |
| Hemoglobin | Normal to  low | 85/1269 (6.7) | 60/910 (6.6) | 65/772 (8.4) | 53/686 (7.7) | 49/618 (7.9) | |
| Leukocytes | Normal to  low | 12/1323 (0.9) | 8/947 (0.8) | 7/799 (0.9) | 5/710 (0.7) | 8/633 (1.3) | |
| Neutrophils | Normal to  low | 15/1231 (1.2) | 9/881 (1.0) | 12/745 (1.6) | 6/662 (0.9) | 11/589 (1.9) | |
| Platelets | Normal to  low | 4/1241 (0.3) | 3/893 (0.3) | 9/752 (1.2) | 10/665 (1.5) | 6/595 (1.0) | |

*Includes all patients in the exposure period with a baseline value and a post-baseline value in the exposure period. ^†^Clinically important shift in direction specified. Proportion of patients with a normal (n) value at baseline shifting to an abnormal (m) value at the last post-baseline measure during the specific exposure period.

ALT = alanine aminotransferase; AST = aspartate aminotransferase.
